# Night science

**DOI:** 10.1186/s13059-019-1800-6

**Published:** 2019-08-28

**Authors:** Itai Yanai, Martin Lercher

**Affiliations:** 1 Institute for Computational Medicine, NYU Langone Health, New York, NY 10016 USA; 2 Institute for Computer Science & Department of Biology, Heinrich Heine University, 40225 Düsseldorf, Germany

If we are woken up in the middle of the night and asked “How does science work?!?”, we rattle off a coherent answer: you have a hypothesis, you use it to make predictions, you test those by comparing them to data, and you throw out or modify the hypothesis if predictions and data disagree. That’s what our teachers taught us. And it’s not wrong. But this description hides the workings of the most exciting part of science, ignoring the most creative and arguably most significant part of our work. By focusing on the structured, rational testing of hypotheses through experiments—*day science*—this description leaves out *night science*, as François Jacob called it. Night science is where we explore the unstructured realm of possible hypotheses, of ideas not yet fully fleshed out. In day science, we falsify hypotheses and observe which are left standing; in night science, we create them. The workings of night science are rarely discussed, as they seem abstract and less concrete compared to the logical description of the formal scientific method. Yet, we believe that there is a method to the madness, and that its conscious study may add an important dimension to our development as scientists. In future installments of this mini-series, we will shed more light on the properties and dependencies of this dark side of science.

We were stuck, and it was extremely painful. The two of us had sacrificed a lot to be there. Both of us were away from home, and isolated from the rest of our scientific community. Somehow we had conspired to find this window of time—a single week—to take a chance on a project we were both excited about. The premise of the project seemed solid enough. In our previous studies [1, 2] we had noticed that gene expression is extremely noisy. And so while we had originally imagined that a gene is turned “on” only when it is needed, it was dawning on us that gene expression may be so noisy that much of it could even be random, in addition to a ‘functional’ program that must surely be under natural selection. Since genome evolution is typically studied in terms of both selected function and random drift, it seemed a good idea to treat gene expression in a similar way: as a set of characters subjected to the vagaries of chance and necessity.

So there we were in Heidelberg 15 years ago, on a mission to explore this idea. A lot of gene expression profiles had been deposited in public databases. We downloaded the data to our laptops and sat in our favorite cafe from morning to night to analyze it together; and no, that wasn’t the painful part. We were not sure exactly what we were looking for, but we were sure something interesting was hiding in that data. Multiple times during the day, we would come up with a plan for a new or modified analysis. We each coded it, one of us in Matlab, one in R. When we would compare notes, we almost inevitably first had different answers, and so we each debugged and compared again until our answers converged. What did the answer mean? We sank back into brainstorming mode, as the results were typically confusing and could be interpreted in any one of several ways. We kept jumping to the question: what was it, precisely, that we were asking? Was this really the right data to tell us about natural selection on gene expression? Periods of rigid work, when we had a specific plan and a specific question to test, alternated with long periods lost in dream worlds, where we conjured up new questions, new ways to tackle the data.

This process bore little resemblance to the scientific method as it had been taught to us: you choose a field, then a problem within that field. You take a set of alternative hypotheses that might solve the problem. Then you collect data that allows you to test the hypotheses. And finally, you may converge on one hypothesis that you cannot rule out—the birth of a theory. But in our projects it just didn’t look like that. We often did not even know what the problem was. All we had were observations that did not seem to make sense based on our expectations. To make sense of them, we tried to examine these observations under the light of evolution. What was the hypothesis? We had no hypothesis. We had no well-defined problem either. This certainly wasn’t the scientific method. So is this just the story of another failed project? We want to argue that it isn’t, and that it instead exemplifies a systematic discordance between the “scientific method” and science as most of us experience it. In fact, many a young scientist’s depression may have its roots in this discordance.

François Jacob, who shared the 1965 Nobel Prize for Physiology and Medicine with André Lwoff and Jacques Monod, had a picture that may capture the full scientific process much better than the current paradigm of “hypothesis-driven” research. In his autobiography, Jacob distinguishes two modes of scientific work, which he referred to as day science and night science [3]. Day science is the one you read about in the news, it is the one we learn about in school, the one captured by the phrase “hypothesis driven”. It’s epitomized by the women and men in white lab coats holding pipettes or looking intently at a computer screen. A day scientist is a hunter who has a clear picture of what she is pursuing.

**Fig. 1:**
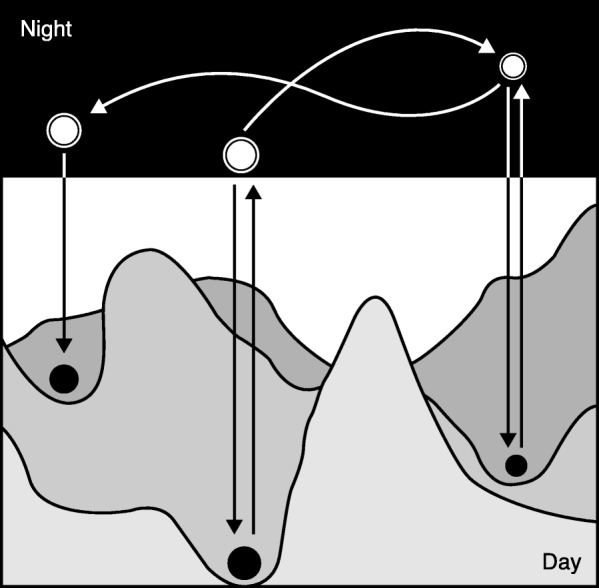
The popping-out model of day science and night science. Day thinking proceeds in logical steps, and thus only ideas that are closely related to the current hypothesis can realistically be reached (symbolized by the isolated valleys in the lower part of the picture). But one can pop out to the much more open night science world, where leaps among ideas are made possible by intuition, associative thinking, unexplained observations, and loosely applied principles from other fields. When a new idea has been generated, one can pop back into the day below and test it efficiently using day science methods

But the bright day is just one half of the cycle. What is on the night side? Reflect for a second on the hypothesis that you are testing. Did you pull it from the ether? How? There is no single answer to this question. In many cases, we may not even have a coherent answer, which may be why we prefer not to include it in most accounts of the scientific process. As Jacob says: “Night science wanders blind. It hesitates, stumbles, recoils, sweats, wakes with a start. Doubting everything, it is forever trying to find itself, question itself, pull itself back together. Night science is a sort of workshop of the possible where what will become the building material of science is worked out” [3]. In day science, we may test a hypothesis using established protocols, and we may move to neighboring ideas in small, logical steps. But ideas that are unconnected or only loosely connected are out of reach when all we rely on are established protocols and logic. This is why we often have to pop out into the world of night science, where we float between ideas that may be only loosely connected, often moving in associative leaps rather than in logical steps (Fig. 1). Intermittently, we may pop back into the world of day science to examine the apparent merit of a night science idea in the light of day, and maybe to even submit it to the rigorous hypothesis testing at the heart of day science—before popping back out into the dream world above to continue our exploration. Night science is of course not restricted to a particular time of day, just as we can test hypotheses after 10 pm. But these two aspects are distinct frames of mind—so different that they seem like day and night.

Night science is a crucial part of science, and the creativity that we find in this realm is not only needed for the generation of novel hypotheses but also, for example, for the development of new methodologies. And yet when we talk about science, we make it sound as though it is a march of pure rationality, where scientists go from one logical step to another. But science as we know it is not like that; its night side is far from clean and rigorous. To cover up this untidy side, and encouraged by journal editors and reviewers, we take great care to tell the stories of our projects as if they had consisted of rigorous day science from day 1, hiding the night from the eyes of our peers. While we do not advocate that all scientific papers should be written as a diary-style account of the actual process, we do believe that the stories of night science are not only beautiful, but that their explicit study would add an extremely important perspective to the nurturing of young scientists. If we told each other about the process of how our hypotheses actually came about, we might all dive more confidently into our next night science explorations. We may even be able to distill strategies for how to make this creative process more fun and productive.

The distinction between executive, systematic day phases and exploratory, creative night phases is not limited to science, and may be general to all enterprises that require at least a minimal level of creativity. In the visual arts, for example, one might distinguish between day art and night art. Day art executes an idea in the studio. Night art is the phase that comes before—or sometimes in between—the execution, where the artist develops the idea of *what* to create—the composition of a painting or a sculpture, for example. By the time the artist knows what she wants to paint, a majority of the creative process may already have happened. In the same way, there may be day music, the act of producing sound or of working out the details of an arrangement, and night music, where musical ideas take shape. In all these fields—science, art, music—the contribution of the creative, night time activity to the success of the whole project is obvious. But there is hardly any human activity that does not require some level of creativity, and so at least a minimal amount of “nightly” exploration may be an integral part of almost everything humans do.

So what about our own experience in the Heidelberg project? No publication ever came out of it, nor any hypothesis that awaits its testing. Does that mean it wasn’t science? Certainly not. Was it a waste of time? No. Those night science explorations were not only fun; together, we explored a region of the night that each of us came back to often in the future, drawing ideas for project proposals and eventually papers [4–9]. In that sense, night science never fails to be productive—we always broaden and reshape our thinking and its horizons. Those explorations put us on the track to formulate the right questions in future projects—a crucial night science activity that we’ll explore in the next installment of this mini-series.
